# Origin and Spread of the ALDH2 Glu504Lys Allele

**DOI:** 10.1007/s43657-021-00017-y

**Published:** 2021-09-20

**Authors:** Xiaokai Zhang, Aijun Sun, Junbo Ge

**Affiliations:** 1 Department of Cardiology, Zhongshan Hospital, Fudan University, Shanghai Institute of Cardiovascular Diseases, Shanghai 200032, China; 2 Institute of Biomedical Sciences, Fudan University, Shanghai 200032, China

**Keywords:** Acetaldehyde dehydrogenase 2, Gene, Environment, Precision medicine

## Abstract

Gene polymorphism of acetaldehyde dehydrogenase 2 (ALDH2), a key enzyme for alcohol metabolism in humans, can affect catalytic activity. The ALDH2 Glu504Lys mutant allele has a high-frequency distribution in East Asian populations and has been demonstrated to be associated with an increased risk of cardiovascular disease, stroke, and tumors. Available evidence suggests that the evolution of the ALDH2 gene has been influenced by multiple factors. Random mutations produce Glu504Lys, and genetic drift alters the frequency of this allele; additionally, environmental factors such as hepatitis B virus infection and high-elevation hypoxia affect its frequency through selective effects, ultimately resulting in a high frequency of this allele in East Asian populations. Here, the origin, selection, and spread of the ALDH2 Glu504Lys allele are discussed, and an outlook for further research is proposed to realize a precision medical strategy based on the genetic and environmental variations in ALDH2.

## Introduction

The acetaldehyde dehydrogenase (ALDH) superfamily consists of enzymes that catalyze the oxidation of acetaldehyde to acidic metabolites, participating in a variety of physiological and pathological processes (Jackson et al. 2011). Nineteen isoenzymes have been identified; the most active one is acetaldehyde dehydrogenase 2 (ALDH2), a 56-kDa tetramer that plays an important role in alcohol metabolism, converting acetaldehyde, an intermediate of alcohol metabolism, to acetic acid (Vasiliou et al. 1999). However, a G to A mutation at rs671 in the ALDH2 gene causes replacement of glutamate with lysine at residue 504, resulting in a reduction in ALDH2 activity, allowing acetaldehyde to accumulate in the body after alcohol consumption, with adverse effects such as flushing and tachycardia (Matsumura et al. 2019).

The frequency of this variant of ALDH2 (ALDH2 Glu504Lys) is low in caucasian, whereas the frequency is 30–50% in East Asian populations, especially in Chinese, Korean and Japanese (Goedde et al. 1992). ALDH2 has been shown to play an important role in a variety of diseases. According to our previous studies, mitochondrial ALDH2 acts as endogenous protection in the heart, and it is closely associated with the development of several cardiovascular diseases (Li et al. 2019). Our studies have demonstrated that patients with ALDH2 Glu504Lys variant are more inclined to suffer from coronary artery lesions (Xu et al. 2018) and displayed a higher risk of developing poor coronary collateral circulation (Liu et al. 2015). Downregulation of ALDH2 in the mitochondria can also lead to cardiomyocyte apoptosis after myocardial infarction in mouse models (Sun et al. 2014). In addition, we have found that ALDH2 deficiency can aggravate energy metabolism disturbance and diastolic dysfunction in diabetic mice (Wang et al. 2016). Moreover, it has been shown that the ALDH2 Glu504Lys variant attenuates bioconversion of nitroglycerin, thus reducing its therapeutic effect on coronary artery disease (Li et al. 2006). In addition, the role of the ALDH2 Glu504Lys variant in stroke (Sun and Ren 2013) and tumors (Suo et al. 2019; Hidaka et al. 2015; Sakamoto et al. 2006) is attracting increasing attention. Therefore, this genetic ALDH2 variant is a serious threat to the health of East Asian and Asian populations, and it may be strongly associated with higher stroke and cardiovascular disease mortality in Asians (Benjamin et al. 2018; Shaw et al. 2008; Zhou et al. 2019).

Given the high frequency of the ALDH2 Glu504Lys allele in East Asian populations, we sought to investigate how this mutation arose, why this deleterious mutation was not eliminated by natural selection, and why it shows high frequency only in East Asia. Here, we discuss the origin, selection and spread of the ALDH2 Glu504Lys allele from an evolutionary perspective and propose some hypotheses in conjunction with previous literature reports, hoping to gain insights into ALDH2 genetic polymorphism to better improve the prevention and treatment strategies in the era of precision medicine.

## Origin of the ALDH2 Glu504Lys Allele

It is now generally accepted that humans originated in Africa (Ingman et al. 2000). Approximately 100,000 years ago, the ancestors of modern humans began to migrate out of Africa and around the world, reaching southern East Asia approximately 50,000–60,000 years ago and then spreading from south to north throughout the region (Palanichamy et al. 2004). Studies examining genetic polymorphisms on the Y chromosome and mitochondrial DNA suggested that various populations underwent multiple divisions and fusions during this migration from Africa to East Asia (Underhill et al. 2001). Since there are rare reports of ALDH2 Glu504Lys in populations of African and European ancestry, we hypothesize that this gene arose after humans migrated from Africa to the rest of the world and originated in the part of African archaic humans that settled in East Asia. Mutations arise through a random process, not an adaptation to the environment, and even the majority of mutations found in experiments are deleterious (Nei 2005). Several point mutations in the ALDH2 gene are produced, but the mutation at the rs671 locus affects enzyme activity, and it became a deleterious mutation. Genetic drift played an important role in the small breeding populations in ancient East Asia; indeed, the populations were sparsely distributed, with considerable distances among populations (Lynch et al. 2016). When a population is small, random mating between individuals causes random fluctuations in allele or haplotype frequencies, such that a certain allele or haplotype will eventually be fixed. The evolution due to genetic drift is random. The genetic variation fixed in a population is independent of the effect of natural selection, and the effect is stronger in smaller populations (Masel 2011).

It has been suggested that the ALDH2 Glu504Lys allele originated from the Pai-Yuei tribe, who was distributed along the southeastern coast of China thousands of years ago (Luo et al. 2009). It has also been suggested that rather than originating in indigenous populations in the region where it now has the highest frequencies, the ALDH2 ∗ 504Lys allele was most likely carried south by Han Chinese immigrants from central China (Li et al. 2009). Although these hypotheses are lack of strong evidence, they suggest that the mutation probably originated in a specific population, namely, an ancient tribe with a small population and the lack of genetic exchange, after which a stable frequency of the mutated gene gradually developed after several generations of genetic drift. In a more extreme case, the founder effect, in which some of the genes of a few ancestors of the population gradually reach high levels due to genetic drift, may occur if the population is completely isolated, without any gene exchange with other populations (Zlotogora 1994).

## Selection of the ALDH2 Glu504Lys Allele

Although the generation of the ALDH2 Glu504Lys allele can be explained by random mutations and genetic drift, these effects on gene polymorphism are very limited and confined to small populations. Hence, there may be some selective forces to maintain its high frequency in East Asia. Indeed, some studies on ALDH2 genetic polymorphism have indicated that the ALDH2 ∗ 504Lys allele had some compensating advantage because they tended to drink less alcohol or had some other advantages (Li et al. 2009; Goldman and Enoch 1990; Lin and Cheng 2002), as in the case of the gene that causes sickle cell anemia conferring a compensating advantage of a certain level of resistance to malaria. Although this issue is currently unresolved, the available evidence suggests that multiple factors may be involved in this process.

Selection theory suggests that natural selection plays a major role in the generation and maintenance of genetic polymorphisms in populations (Nei 2005). It is worth noting that natural selection does not select for health but only for successful reproduction; thus, a gene will be retained even if it is detrimental to the health of the individual, as long as it does not reduce the number of surviving offspring. Therefore, whether the ALDH2 Glu504Lys allele is retained depends on whether the mutation affects the number of surviving offspring under certain external environmental conditions.

### Relationship with Alcohol Consumption

Alcohol consumption behavior is one of the environmental factors most closely associated with ALDH2. The ALDH2 Glu504Lys allele not only causes individuals to experience adverse reactions such as blushing after alcohol consumption, but it also increases the risk of heart disease (Shen et al. 1863), esophageal cancer (Suo et al. 2019), gastric cancer (Hidaka et al. 2015), liver cancer (Sakamoto et al. 2006), and other diseases in alcohol drinkers. Nevertheless, during the evolution of the ALDH2 gene, alcohol consumption did not eliminate the deleterious mutation, i.e., Glu504Lys, by negative selection or even reduce its frequency. Although the exact reason for this remains unknown, it may be because wine originated after this mutation and the ALDH2 Glu504Lys allele was stable in the population before humans learned to produce wine. In addition, as large-scale brewing was available in ancient times, alcohol consumption was low, and the amount produced was small. Therefore, the disadvantages of the mutated gene were not obvious in such an environment of a relatively low frequency of alcohol consumption and low amounts. Furthermore, the ALDH2 Glu504Lys allele can even prevent individuals from drinking due to the “blushing syndrome” and other adverse reactions. As we know, some genes that were beneficial or at least harmless in the ancient environment can cause problems in the modern world. Just as the “thrifty genes” would be advantageous under the conditions of unpredictably alternating feast and famine that characterized the traditional human lifestyle, but they would lead to obesity and diabetes in the modern world (Diamond 2003). In conclusion, the effect of alcohol consumption on individuals carrying the ALDH2 Glu504Lys allele would not likely affect the number of surviving offspring or eliminate them through selection, at least in the ancient environment.

### Relationship with Hepatitis B Virus Infection

It has been suggested that hepatitis B virus infection plays a selective role in this process, with the ALDH2 Glu504Lys allele being evolutionarily advantageous for hepatitis B virus carriers (Lin and Cheng 2002). In terms of geographical distribution, the infection rate of hepatitis B virus in East Asia is significantly higher than that in Europe and America (Schweitzer et al. 2015); in China, the rate is higher in the southeast coastal region and lower in the western region (Beasley et al. 1982). Hence, there may be a geographical correlation between HBV prevalence and ALDH2 deficiency within China and some other places to some degree. Studies have shown that the ALDH2 Glu504Lys allele increases the risk of liver cancer in alcohol drinkers (Sakamoto et al. 2006; Abe et al. 2015), but no increased risk is observed in non-drinkers (Liu et al. 2016). As both alcohol consumption and hepatitis B virus infection are the risk factors for liver cancer, those carrying the ALDH2 Glu504Lys allele may reduce alcohol consumption among hepatitis B virus-infected individuals, indirectly reducing their risk of developing liver cancer (Liu et al. 2016). In addition, the ALDH2 Glu504Lys allele may attenuate liver damage in alcohol drinkers, with reduced AST, ALT, and GGT levels observed in these individuals (Takeshita et al. 2000). The mechanism may be explained by weakened acetaldehyde metabolism, reduced NADH production and oxidative stress (Matsumoto et al. 2007), as well as inhibition of NF-kB and MAPK pathways, decreased TNF-a production, and attenuated hepatic inflammatory response by increased ethanol load (Matsumoto et al. 2008; Lindros et al. 1999). Thus, on the one hand, the ALDH2 Glu504Lys allele alleviates liver damage in hepatitis B virus-infected individuals; on the other hand, it reduces the risk of liver cancer by reducing alcohol consumption, thus conferring these individuals with a better chance of survival. Hepatitis B virus infection may also exert a positive selection effect on the ALDH2 Glu504Lys allele, favoring its retention. In particular, individuals heterozygous for ALDH2 Glu504Lys may exhibit a heterozygote advantage, experiencing both a hepatoprotective effect not found in those homozygous for the wildtype gene and higher enzyme activity than in those homozygous for the mutant, which may allow the allele to reach a high frequency in some populations. A classic case is that the high frequency of the sickle cell haemoglobin (HbS) gene in malaria-endemic regions is thought to be related to a heterozygote (HbAS) advantage against fatal malaria. It has been shown that heterozygotes (HbAS) can provide significant protection against all-cause mortality and severe malarial anaemia compared with HbAA genotypes (Aidoo et al. 2002).

### Relationship with High-Elevation Hypoxic Environments

Another possible selection factor is related to oxygen concentrations. ALDH2 has been shown to be essential for tolerance to hypoxic conditions, and it plays a protective role in hypoxia-related diseases such as ischemic heart disease (Liu et al. 2015; Sun et al. 2014) and hypoxic pulmonary hypertension (Zhao et al. 2019). Although the ALDH2 Glu504Lys allele has a high frequency in East Asia in general, it is less frequently distributed in the Tibetan population (4.4%) than in the Han populations of Guangdong (24.8%), Qingdao (17.6%), and Liaoning (12.2%) (Luo et al. 2009), and this difference may be related to the low oxygen environment of the plateau in Tibet. After the divergence of the common ancestors of the Han and Tibet populations in the early Neolithic period, the Han people migrated eastward into the plains, whereas Tibetans settled westward in the plateau region (Shi et al. 2005). Negative selection of the ALDH2 Glu504Lys allele under the low oxygen environment may have occurred in this population entering Tibet, resulting in its reduced frequency. In contrast, the ALDH2 Glu504Lys allele maintained a higher frequency in the Han population living in the plains, especially in the densely vegetated and humid southeastern coastal zone, because its disadvantage was not revealed due to sufficient oxygen in the environment.

In addition, a higher rate of EGLN1 gene mutation has been demonstrated in Tibetans, reducing EGLN1 activity and facilitating adaptation to hypoxic conditions (Simonson et al. 2010). It was shown that ALDH2 activity in isolated hepatocytes of EGLN1-deficient mice was increased by 25% compared to wildtype and that levels of ethanol metabolism and ROS (reactive oxygen species) clearance were increased (Laitakari et al. 2019). It has been suggested that the hypoxic environment may also indirectly affect the activity of ALDH2 through a selective effect on the EGLN1 gene. The population in the plains is lack of EGLN1 gene mutation, and if they carry the ALDH2 Glu504Lys allele, it will be more difficult for them to adapt to the hypoxic environment in the Tibetan plateau. However, studies to date have not resolved this issue, and the relationship remains to be proven by further evidence.

In conclusion, it is difficult to obtain a clear explanation for the evolutionary mechanism of the ALDH2 gene based on the findings thus far, and multiple factors may have played a role in the selection of the ALDH2 Glu504Lys allele. Ohta proposed the near-neutrality theory of evolution, in which mutation, genetic drift, and selection act simultaneously in evolution (Ohta 2002), which may be able to explain the evolution of ALDH2. A random mutation produced the ALDH2 Glu504Lys allele, genetic drift altered the gene frequency of this gene in the initial small population, and natural selection caused its differental distribution in different environments.

### Dispersal of the ALDH2 Glu504Lys Allele

Since the Neolithic period, populations around the world have been undergoing continuous migration and gene exchange (Yang and Fu 2018), and the spread of the ALDH2 Glu504Lys allele may also be associated with the migration of East Asian populations. Studies have shown that evolution in East Asia has been continuous since *Homo erectus*, and no large-scale replacement of native populations by foreign populations has occurred during this time. Although a small amount of integration of foreign populations with native subject populations occurred, this exchange of genes is minor compared to the intergenerational transmission of native populations (Yang and Fu 2018). This would explain why the spread of the ALDH2 Glu504Lys allele was confined to East Asia, without contributing to the human gene pool outside Asia. Two-way interaction and integration of northern and southern populations in East Asia began in the early Neolithic, and three southward migrations occurred, allowing the spread of East Asian populations to Southeast Asia and the Southwest Pacific islands (Zhang and Fu 2020). The ALDH2 Glu504Lys allele present in Thai and Cambodian populations (Goedde et al. 1992; Oota et al. 2004) may be derived from these migrations; the ALDH2 Glu504Lys allele also has a high frequency in South Korea and Japan (Goedde et al. 1992; Luo et al. 2009). Y-chromosome polymorphism suggests that the Japanese population originated from at least two migrations from mainland Asia (Hammer et al. 2006), and the Korean population may have originated from Northern Asian settlement and range expansion mostly from southern-to-northern China (Jin et al. 2003). In conclusion, Neolithic and subsequent East Asian population migrations played an important role in the spread of ALDH2 Glu504Lys alleles. Although the exact correlation between the migrations of East Asian populations and the dispersal of ALDH2 Glu504Lys alleles have not been confirmed, the distribution of the ALDH2 Glu504Lys allele after this event is quite similar to that of the present day.

## Perspective

Most diseases are caused by a complex combination of genetic and environmental factors, and the causative genetic factors are largely associated with the significant influence of environmental adaptations in human evolution. The ALDH2 gene, an important genetic factor, is not only associated with the risk of digestive tumors associated with alcohol consumption (Suo et al. 2019; Hidaka et al. 2015; Sakamoto et al. 2006) but also contributes to the prevention of alcohol abuse (Shen et al. 1863), ischemia (Liu et al. 2015; Sun et al. 2014), metabolic disorders (Wang et al. 2016), and other adverse factors causing cardiac dysfunction. Although the evolution of the ALDH2 gene has not been elucidated, environmental factors, one of the most important drivers of evolution, undoubtedly played an important role in this process. Therefore, studying the interaction of the ALDH2 gene with specific environments and contexts can help to achieve precision medicine based on genetic and environmental factors for specific populations. For patients with cardiovascular diseases, we should examine their ALDH2 genotype and living environment, whether they have consumed alcohol for a long time or live in high-elevation hypoxic conditions to achieve a more personalized and precise treatment plan. For individuals with ALDH2 mutations, several therapies have been shown to increase the activity of the enzyme (Li et al. 2020; Sun et al. 2017). Therefore, in the future treatment of cardiovascular and other diseases, we may expect to implement ALDH2-based precision medical strategies to improve the health status of people in East Asia and even the global populations.

## Data Availability

Not applicable.
